# Effects of Malarial Poisoning on the Dental Pulp

**Published:** 1884-02

**Authors:** J. A. Klump

**Affiliations:** Harrington, Delaware.


ARTICLE VI.

### THE EFFECTS OF MALARIAL POISONING ON THE DENTAL PULP.

BY J. A. KLUMP, D. D. S., M. D., HARRINGTON, DELAWARE.

(Read before the Pennsylvania State Dental Society, August 1, 1883.)

It is not my expectation in this paper to present to this society any special information with regard to the effects of malarial poisoning on the dental pulp, but to elicit a discussion of the subject. I will endeavor to suggest a few points the consideration of which may interest and purfittos.

We claim as our chief object the salvation of natural teeth. The rapid advancement in dental science is most encouraging to us. Especially are the restorations of badly diseased teeth of to-day remarkable in contrast to our want of success in such cases a few years since. Our endeavors to place these organs in conditions most favorable to resist deleterious agencies are meeting with more success, and our methods are becoming more effective. The value of constitutional treatment is more appreciated, but is worthy of more attention than it has yet received. That something more than local treatment is required is consequently becoming more evident. Local treatment alone will, in many cases prove a failure, but with general treatment combined, will give the desired result. There are cases where nothing but constitutional treatment will be effective. The subject of improving teeth of low vitality by putting into the system the elements in which such teeth are deficient, has been repeatedly discussed, and time and again have we witnessed such cases improve under this treatment: yet, in many instances, no benefit seems to accrue, and until we go deeper and take into account the important functions that preside over secretion, the normal performance of which is necessary for the assimilation of such elements, we may expect many failures. To look after the state of the nervous system, the blood, and the digestive organs, and have their functions properly performed, would be doing more, in many cases, for the health of the teeth than would be accomplished by administering the phosphates, etc., and risking their assimilation.

Especially should the nervous system receive attention. It has an enormous influence over the teeth, controlling circulation, secretion, and nutrition. Not only do general nervous derangements affect the highly sentient pulp of a tooth in general proportion to the nervous tissue involved, but, owing to its peculiar situation,—incased with a dense wall of dentine,—nervous phenomena which produce the slightest irregularities in the circulation will cause prominent symptoms of irritation here. The blood vessels are subject to dilatation, and the surrounding tissues, principally nervous, must suffer compression in proportion to the amount of congestion. Considering the fact that death of the pulp in the majority of cases is due to strangulation, it becomes essential in its conservative treatment to combat all causes predisposing to congestion. Malarial poisoning being productive of marked irregularities in the distribution of the blood supply, and, moreover, creating serious nervous disturbance, and tending to a general lowering of the entire organism, its influence should be combated, in view of its effect upon the teeth. Irritation of the pulp is frequently the initial symptom of malarial impression, and not infrequently is of sufficient severity to cause death of the pulp in sound teeth, which is a common occurrence in highly malarious sections. That death of the pulp may be due to malarial poisoning is assumed, because it occurs in subjects suffering from malaria, and when there is no other evident cause for the irritation; and the majority of cases of pulp irritation under such circumstances yield promptly to early treatment with quinia. In the commencement of miasmatic fevers, it is not uncommon to have odontalgia of a neuralgic character, which is generally recognized by physicians as being of malarial origin, and they treat it as they would malarial pains of any part, by bringing about "cinconsism" as quickly as possible. We may at times encounter cases of sensitive dentine of malarial causation, in which local obturndents have little if any effect. These cases are usually associated with general nervous excitability, and the mere touch of an excavator to the tooth will so unnerve the patient as to render an operation very unsatisfactory, if not impossible of performance. Here three or four grain doses of quinia, or the equivalent, of one of its alkaloids, given every four hours, until about thirty grains have been administered in anticipation of the sitting, will not only allay the sensibility of the dentine, but will produce such a quieting effect on the general nervous system that operations can then be performed with satisfaction to patient and operator. In these cases nothing but anti-malarial treatment will have much effect. The simple operation of capping is often, the presence of malaria in the system, rendered very uncertain. This will account for the success of some practitioners in this operation; while others are ready to condemn it on account of its uncertainty. Between the month of June and the frosts af autumn, when this disturbing element prevails, we have, doubtless, all experienced its influence more or less in varying results from like treatment in different cases. In teeth successfully capped under favorable circumstances, trouble is subsequently excited by this agent.

In the personal experience of the writer, recently located in the malarial climate of Delaware, this has been forcibly demonstrated. In a tooth capped and filled in 1880 there was no discomfort experienced until the present summer, when, during an attack of remittent fever, there was an intense irritation of the pulp, which subsided in a few hours, cinchonism having been established in the treatment of the fever. The symptoms once recurred in a mild degree, coincident with the reappearance of positive symptoms of the malarial trouble. Case after case similar to this has come under my notice. A capped pulp, or the pulp of a filled tooth is especially liable to malarial disturbance—thermal changes doubtless assisting in a measure in exciting irritation. Or, the pulp being less vigorous on account of its artificial surroundings, yields more readily than in health. It is a recognized fact that congestion is liable to involve a weaker organ. Where there is a congestive tendency, a trifling irritation only is necessary to determine it to a given point.

As to the manner in which malaria excites irritation in the pulp we can only speculate. We know that in subjects saturated with malaria the blood is found to have undergone a marked change; the red blood corpuscles are diminished in number; the blood is thinner than normal, and pigment matter is frequently in such excess as to cause thrombi in the smaller capillaries. The walls of the blood vessels lose their tenacity, thus favoring the effusion of their contents. The function of the spleen, as a diverticulum for the blood is frequently interfered with, thus depriving the congested capillary system of the relief which a proper performance of the splenic function affords. The parasite which has been found in the red blood corpuscles of malarial patients may contribute to the formation of thrombi, or its presence may be capable of establishing organic disease of the pulp, as it is believed to excite disease of the kidney, liver, etc., by its mere presence within these organs. From the known facts as to the nature of malarial poisoning, it is probable that the circulation in the pulp must be influenced by it in no small degree. The contracted apex of the root of a tooth is highly favorable to the formation of a thrombus within the pulp chamber, while the blood pressure neccssary for forcing its return from the pulp circulation would favor effusion into the pulp tissue—a result which we know would prove disastrous to a living pulp. The parasite may excite irritation from pressure if sufficient to occlude the blood vessels, or their presence as a foreign body may produce irritation of the sensory nerves sufficient to bring about extreme congestion.

It cannot be doubted that malaria has a deleterious influence on the pulp of a tooth and on the health of tooth structure generally, and it should certainly receive the importance it demands.—*Dental Practitioner.*

